# Insulin Requirement Profiles of Short-Term Continuous Subcutaneous Insulin Infusion Therapy in Patients With Type 2 Diabetic Nephropathy

**DOI:** 10.1155/ije/8403917

**Published:** 2025-03-13

**Authors:** Qianqiu Feng, Biyun Li, Jiating Lin, Li Zou, Wen Xu, Longyi Zeng, Ping Li, Shuo Lin

**Affiliations:** ^1^Department of Endocrinology & Metabolism, The Third Affiliated Hospital of Sun Yat-Sen University, Guangzhou 510630, Guangdong, China; ^2^Department of Endocrinology & Metabolism, Heyou Hospital, Shunde 528306, Foshan, China; ^3^Department of Endocrinology & Metabolism, The Affiliated Changsha Central Hospital, Hengyang Medical School, University of South China, Changsha 410000, China; ^4^Guangdong Provincial Key Laboratory of Diabetology, The Third Affiliated Hospital of Sun Yat-Sen University, Guangzhou 510630, Guangdong, China; ^5^Guangzhou Municipal Key Laboratory of Mechanistic and Translational Obesity Research, The Third Affiliated Hospital of Sun Yat-Sen University, Guangzhou 510630, Guangdong, China; ^6^Department of Obstetrics, The Third Affiliated Hospital of Sun Yat-Sen University, Guangzhou 510630, Guangdong, China

## Abstract

**Objective:** This study aimed to explore the insulin requirement during continuous subcutaneous insulin infusion (CSII) treatment in patients with type 2 diabetic kidney disease (T2DKD).

**Methods:** This study retrospectively analyzed the clinical data of 150 T2DKD patients in the Department of Endocrinology and Metabolism of the Third Affiliated Hospital of Sun Yat-sen University from January 2018 to December 2021. All patients received short-term CSII treatment and achieved the blood glucose target by adjusting insulin infusion. The patients' daily insulin requirements during the treatment were recorded and analyzed.

**Result:** There were 91 males and 59 females with an average age of 60.6 ± 10.6 years. Total daily insulin dose (TDD), total daily insulin dose per kilogram (TDD kg^−1^), total basal insulin dose per kilogram (TBa kg^−1^), and total bolus insulin dose per kilogram (TBo kg^−1^) fell with the decline of eGFR when achieving the blood glucose target except for the ratio of total basal insulin dose (TBD) to TDD (%TBa). Insulin requirement was less in patients with eGFR < 60 mL/min/1.73 m^2^ compared to those with eGFR ≥ 60 mL/min/1.73 m^2^ (*p* < 0.05). When achieving the blood glucose target, eGFR ≥ 60 mL/min/1.73 m^2^ group and eGFR < 60 mL/min/1.73 m^2^ group were 0.71 ± 0.19 U/kg and 0.60 ± 0.24 U/kg, respectively. Multiple linear regression analyses showed that glycated hemoglobin and eGFR were independent factors associated with TDD kg^−1^.

**Conclusion:** In patients with T2DKD who received short-term CSII therapy, the insulin requirement decline with the decrease of eGFR, while the %TBa did not change significantly. The dosage of insulin should be adjusted according to the level of eGFR in patients with T2DKD treated with CSII.

## 1. Introduction

Diabetic nephropathy (DN), also referred to as diabetic kidney disease (DKD), is a common microvascular complication of diabetes worldwide. The incidence of DN has been increasing significantly each year with the rising prevalence of diabetes, causing around 30%–50% of cases of End-Stage Renal Disease (ESRD) globally [1]. In China, DN has become the leading cause of ESRD among middle-aged and elderly individuals [2]. Although early, intensified blood glucose control can delay the progression of DN to a certain extent, impaired kidney function can lead to the accumulation of exogenous insulin and oral hypoglycemic drugs in the body, increasing the risk of hypoglycemia. Therefore, the optimal strategy for DN management should focus on effectively reducing blood glucose levels without increasing the risk of hypoglycemia.

Continuous subcutaneous insulin infusion (CSII) has been studied as a possible therapy for type 2 diabetes (T2DM) for over 30 years, which helps to rapidly reduce hyperglycemic toxicity and improve patient outcomes, and is one of the important means of short-term insulin-intensive therapy. CSII can not only improve islet beta cell function in newly diagnosed T2DM patients [3] but also has been reported to restore islet beta cell function in some patients with longer T2DM [4]. Recently, short-term CSII therapy has been widely applied in inpatient patients with T2DM, particularly in China [5].

At present, there have been many studies on the use of CSII in newly diagnosed T2DM patients, but there are still few literature reports on the application of CSII in diabetic patients with chronic complications. While some studies have found that appropriately reducing basal and mealtime insulin doses in T2DKD patients undergoing multiple daily injection (MDI) therapy can decrease the incidence of hypoglycemia without affecting glycemic control, there are currently no reports on the insulin dose characteristics used in CSII therapy for T2DKD patients. In clinical practice, the insulin dosage used in CSII and its subsequent titration depend highly on the doctor's experience. Lack of sufficient evidence may lead to slow achievement of blood glucose targets or the occurrence of hypoglycemic events. In previous small sample retrospective study, we found that the ratio of basal insulin dose to total daily insulin dose (TDD) might be decreased while eGFR declined in T2DKD patients [6]. Therefore, we conducted this retrospective study to analyze the insulin dose characteristics of T2DKD patients undergoing short-term CSII therapy in different stages of renal disease and to analyze the impact of combined oral hypoglycemic drugs on insulin dosage during CSII therapy.

## 2. Materials and Methods

Clinical data of 150 subjects diagnosed as T2DKD (mean age 60.6 ± 10.6 years, 60.7% were male) who received short-term CSII treatment during hospitalization in the Endocrinology Department of the Third Affiliated Hospital of Sun Yat-sen University from January 2018 to December 2021 were retrospective analyzed. Subjects were hospitalized for glycemic control and complications' evaluation. Patients with severe infections, acute complications (including diabetic ketoacidosis and hyperosmolar hyperglycemic state), decompensated heart failure, severe liver diseases, non-DKD, those currently receiving corticosteroid therapy and blood dialysis, were excluded. The present study was approved by the ethics committee of the Third Affiliated Hospital of Sun Yat-sen University. Written informed consent was obtained from all participants before the survey.

Fasting for 10 h, weight, height, body mass index (BMI), waist circumference, and blood pressure (BP) of the subjects were measured on the next morning after admission. Blood was drawn for testing glycosylated hemoglobin (HbA1c), fasting C-peptide level, fasting blood glucose (FPG), blood creatinine (Cr), eGFR (CKD-EPI), and hemoglobin level (HB), and the urine albumin/creatinine ratio (UACR) were measured. Subsequently, patients were treated with rapid-acting insulin analogs, including insulin aspart or insulin lispro by using Medtronic (712 or 722) insulin pumps. The initial insulin dose was given as a TDD of 0.4–0.8 IU/kg, with 40%–50% allocated to the total basal dose and 50%–60% allocated to the bolus dose. The initial basal dose was divided into six time periods: from 00:00 to 03:00, from 03:00 to 07:00, from 07:00 to 12:00, from 12:00 to 17:00, from 17:00 to 22:00, and from 22:00 to 24:00, according to the basal dose table. The total bolus dose was evenly distributed before each meal. Insulin doses were adjusted in a timely manner based on capillary blood glucose measurements (glucose dehydrogenase method) to achieve the target blood glucose level. Blood glucose targets were set at 4.4–7.0 mmol/L for fasting capillary blood glucose and 4.4–10.0 mmol/L for 2 h postprandial capillary blood glucose. Hypoglycemia was defined as capillary blood glucose concentration lower than 3.9 mmol/L, with or without hypoglycemia symptoms. If a patient's blood glucose was poorly controlled during CSII treatment, physicians would use oral hypoglycemic drugs based on experience to better control blood glucose. Within 1–2 weeks of hospitalization, all patients received diabetes self-management education, including diabetes diet guidance, exercise recommendations, lifestyle adjustments, and food intake guidance. The daily total calories of diabetes diet were 25–30 kcal/standard kilogram of body weight, distributed as 1:2:2 for three meals, and additional food or drink was not recommended unless to correct hypoglycemia. During CSII treatment, patients' insulin requirement profiles were recorded daily. The total insulin dose, basal insulin dose, and bolus insulin dose, as well as relevant clinical factors, were analyzed on the first day of achieving blood glucose target and on the last day of intensive treatment. The study population were divided into four groups according to eGFR: G1 group (≥ 90 mL/min/1.73 m^2^), including 37 patients; G2 group (≥ 60 mL/min/1.73 m^2^ and < 90 mL/min/1.73 m^2^), including 39 patients; G3 group (≥ 30 mL/min/1.73 m^2^ and < 60 mL/min/1.73 m^2^, including 37 patients; G4–5 group (< 30 mL/min/1.73 m^2^), including 37 patients (Supporting Table 1).

Statistical analysis was performed by using the SPSS 25.0 software. Normally distributed quantitative data were expressed as mean ± standard deviation ($\bar{x}$ ± *s*), and the two independent sample *t*-test was used for comparison between two groups. ANOVA was used for comparison among multiple groups, and the LSD method was used for pairwise comparison between groups. Non-normally distributed quantitative data were expressed as median (interquartile range), and the Mann–Whitney test was used for comparison between two groups. The Kruskal–Wallis test was used for comparison among multiple groups. Chi-square test and Fisher's exact probability test were used for comparison of count data between groups. Pearson correlation (for normally distributed data) and Spearman correlation (for non-normally distributed data) and multiple stepwise linear regression were used to evaluate the correlation between insulin dose and other variables. A *p* value < 0.05 was considered statistically significant.

## 3. Results

### 3.1. Clinical Characteristics of the Study Population

A total of 150 patients with T2DKD were included. There were 91 males and 59 females with an average age of 60.6 ± 10.6 years. The median course of disease was 120 months. Seventy-nine cases were combined with oral hypoglycemic drugs. There were significant differences among groups of T2DKD in terms of age (*p* ≤ 0.001), disease duration (*p*=0.002), usage of oral antidiabetic drugs (*p*=0.004), fasting C-peptide (0.010), glycated hemoglobin (*p*=0.011), uric acid (*p*=0.009), serum creatinine (*p* ≤ 0.001), eGFR (*p* ≤ 0.001), and urinary albumin-to-creatinine ratio (*p* ≤ 0.001) (Table 1).

### 3.2. Insulin Dosage Characteristics in Different Subgroups of the Study Population

There were statistically significant differences between groups in insulin dosage among different subgroups of T2DKD subjects, except for the index involving the total bolus insulin dose per kilogram (TBo kg^−1^) and ratio of total basal insulin dose (TBD) to TDD (%TBa). In each group of kidney disease, TDD, TDD kg^−1^, and total basal insulin dose per kilogram (TBa kg^−1^) at the time of achieving blood glucose target showed a downward trend with the decrease of eGFR, and the overall comparison differences were statistically significant. At the end of the study, TDD and TDD kg^−1^ also showed a downward trend with the decrease of eGFR, and the overall intragroup comparison differences were statistically significant (Table 2). Compared to the G1 group, the TDD, TDD kg^−1^, TBa kg^−1^, and TBa kg^−1^ in G3 and G4–5 groups at the time of achieving blood glucose target were decreased (all *p* < 0.05).

### 3.3. Comparison of Insulin Requirement Profiles Between T2DKD Patients With eGFR ≥ 60 mL/min/1.73 m^2^ and eGFR < 60 mL/min/1.73 m^2^

Subjects with T2DKD were divided into two groups by a cut-point of eGFR 60 mL/min/1.73 m^2^. eGFR ≥ 60 mL/min/1.73 m^2^ was defined as Group A, and eGFR < 60 mL/min/1.73 m^2^ as Group B. Most of the insulin dosage in Group B was less than those in Group A. When achieving the blood glucose target, the TDD in Group B decreased by 17.72% compared to Group A (*p*=0.001), the TDD kg^−1^ reduced by 15.49% (*p*=0.003), the TBa kg^−1^ decreased by 15.63% (*p*=0.016), and the TBo kg^−1^ decreased by 12.82% (*p*=0.017), respectively. At the end of the treatment, the TDD in Group B decreased by 15.43% compared with Group A (*p*=0.006), the TDD kg^−1^ decreased by 13.04% (*p*=0.013), the TBa kg^−1^ decreased by 16.13% (*p*=0.035), and the TBo kg^−1^ decreased by 10.53% (*p*=0.047), respectively (Table 3).

### 3.4. Comparison of Insulin Dose Characteristics Between the Single Use of the CSII Group and the Combination Use of OADs Group in Patients With T2DKD

Among the patients with T2DKD, a total of 74 patients were administrated a combination of oral hypoglycemic drugs and CSII, with the highest number of patients (36, 48.65%) using metformin in combination. The second most common drugs used were DPP-4 inhibitors (17, 22.97%) and alpha-glucosidase inhibitors (8, 10.81%). In addition, there were also patients who took two types of oral hypoglycemic agents simultaneously, with the combination of metformin and alpha-glucosidase inhibitors being the most common (5, 6.76%). The comparison of the insulin dosage characteristics between the CSII monotherapy group and the OADs combination group in T2DKD patients revealed that, except for the TBa kg^−1^ in gGroup G2, the TDD kg^−1^ in Group G3 and TBo kg^−1^ in Group G3 (*p* value < 0.05), there was no statistically significant difference in the other indicators (all *p* value > 0.05) (Table 4).

### 3.5. The Independent Associated Factors of TDD Kg^−1^ When Achieving Blood Glucose Target in T2DKD Patients

In the T2DKD population, multiple linear regression analysess were performed with TDD kg^−1^ when achieving the blood glucose target as the dependent variable and age, whether or not combined with OADs, BMI, TC, TG, HDL-C, LDL-C, hemoglobin, fasting C-peptide before treatment, glycosylated hemoglobin, and eGFR as independent variables. The results demonstrated that after adjusting for age, whether or not combined with OADs, BMI, TC, TG, HDL-C, LDL-C, hemoglobin and fasting C-peptide before treatment, glycosylated hemoglobin, and eGFR remained independent associated factors of TDD kg^−1^ when achieving the blood glucose target.

The final multiple linear regression equation was TDD kg^−1^ = 0.343 + 0.002HbA1c% + 0.023⁣^∗^eGFR (*F* = 11.751, *p* ≤ 0.001) (Table 5). The variation of 15.9% in the dependent variable, TDD kg^−1^ when reaching the standard, could be explained by the glycation and eGFR levels (adjusted *R*2 = 0.146). The partial regression coefficients *β* and 95% confidence intervals of each independent variable are presented in Table 5.

The correlation between whether or not to combine with OADs and TDD kg^−1^ when achieving the blood glucose target was not significant (*r* = 0.13, *p*=0.115). No interaction between GFR and use of oral agents had been observed for TDD kg^−1^(*F* = 1.456, *p*=0.591).

### 3.6. Incidence of Hypoglycemia Events in T2DKD Population in Different Renal Function Groups

The occurrence of hypoglycemic events was analyzed among different groups to explore the potential differences (Table 6). The results indicated that the incidence of hypoglycemia varied significantly among the different groups (*p*=0.007). Specifically, the hypoglycemia rate was significantly higher in Group G3 compared to Group G1 (*p*=0.002) (Table 6).

## 4. Discussion

Poor blood glucose control is an obvious risk factor for microalbuminuria and has been extensively studied. Strict blood glucose control is of great significance for the prevention and delay of DKD [7]. However, there are no literature reports on the characteristics of insulin dose in T2DKD patients treated with CSII, and the adjustment of insulin dose depends largely on the experience of the doctors in clinical practice. In this study, we found that in T2DKD patients treated with CSII, the total insulin dose/kg body weight, basal insulin dose/kg body weight, and bolus dose/kg body weight were all decreased with the decrease of eGFR. This result is also supported by some guidelines or consensus. To our knowledge, it is the first study to explore the insulin profiles among T2DKD patients treated with CSII.

The Blood Glucose Safety Committee of Duke University Medical Center in the United States recommends that insulin doses should be reduced by 30%, 50%, and 60%, respectively, for CKD G3, G4, and G5 patients [8]. Another consensus suggests [9] that when eGFR drops to 10–50 mL/min/1.73 m^2^, insulin should be reduced by 25%, and when eGFR < 10 mL/min/1.73 m^2^, it should be further reduced by 50%. However, for CKD G1 ∼ 2 DM patients, insulin usage dose adjustment is unnecessary. Biesenbach et al. [10] conducted a retrospective study that included T1DM and T2DM patients undergoing insulin treatment (MDI) and found that insulin requirements decreased progressively from CKD G1 to G5: T1DM patients dropped from 0.72 U kg^−1^∙d^−1^ to 0.45 U kg^−1^∙d^−1^, and T2DM patients decreased from 0.68 U kg^−1^∙d^−1^ to 0.33 U kg^−1^∙d^−1^. The recommended insulin dosage for kidney disease patients and related research data mentioned above all use the MDI treatment method, and there is no literature reporting the use of CSII. In the present study, we found that the total insulin/body weight, basal dose/body weight, and bolus dose/body weight showed a decreasing trend with a decrease in eGFR. The insulin requirements in T2DKD patients decreased from 0.74 U kg^−1^∙d^−1^ in the G1 group to 0.58 U kg^−1^∙d^−1^ in the G4–5 group. When eGFR drops below 60 mL/min/1.73 m^2^, the insulin required is reduced by 15.49% compared to T2DKD patients with eGFR greater than 60 mL/min/1.73 m^2^. Our results are consistent with the above studies and consensus. Therefore, when applying the CSII treatment to T2DKD patients, the insulin pump dose should be adjusted according to the glomerular filtration rate. Clinical doctors should adjust the insulin dose based on the degree of kidney function impairment when the eGFR drops below 60 mL/min/1.73 m^2^ to achieve blood glucose control goals as soon as possible while closely monitoring blood glucose and preventing hypoglycemia.

It has been demonstrated that approximately 50% of insulin is secreted under basal conditions in healthy individuals without diabetes [11]. Therefore, the formula (TBD = 0.5 × TDD) has been widely recommended for setting the TBD in the CSII treatment to mimic normal insulin secretion. However, a study in 2019 involving 327 T2DM patients receiving CSII treatment in China confirmed a basal insulin requirement of about 40% of TDD [12]. The same %TBa has also been proposed in two other studies involving Asian T2DM patients, which may be related to the high carbohydrate intake and significant postprandial blood sugar elevation in East Asian populations [13, 14]. The latest Chinese CSII treatment guidelines have also been modified based on this, setting the proportion of basal infusion to total dose at 40%–60% (with an average of 50%). In our study, the %TBa was 45%–47% in T2DKD patients, and the %TBa in different eGFR groups showed no statistical difference (*p* > 0.05). Further study is needed to determine whether T2DKD patients in different eGFR groups require different basal insulin dosages.

Currently, it has been proven that the combination therapy of oral hypoglycemic agents and insulin can alleviate insulin resistance, reduce insulin dosage, avoid iatrogenic hyperinsulinemia, reduce the incidence of hypoglycemia, and achieve better glycemic control. In 2018, Li found through the analysis of two randomized controlled trials that the addition of metformin to CSII treatment can significantly reduce the insulin dose required for T2DM patients to control glycemic fluctuations [4]. There are also reports that compared with using CSII alone, the insulin requirements of T2DM patients in the CSII combined with the troglitazone group decreased by 53%, and those in the CSII combined with the metformin group decreased by 31% [15]. Part of subjects in our study received the CSII combined with OADs therapy. We further analyzed that the impact of OADs on the insulin dosage characteristics of CSII T2DKD patients. They were divided into the CSII monotherapy group and CSII combined with the OADs group, and there was no significant difference in most of indicators between the combined with OADs group and the CSII monotherapy group. After adjusting for age, whether or not combined with OADs, BMI, TC, TG, HDL-C, LDL-C, hemoglobin and fasting C-peptide before treatment, glycosylated hemoglobin, and eGFR remained independent associated factors of TDD kg^−1^ when achieving the blood glucose target. Therefore, our results indicated that the insulin dosage of T2DKD patients using CSII therapy decreases with decreasing eGFR, regardless of whether combined with OADs or not. The reason why the use of oral hypoglycemic agents did not affect insulin dosage significantly in this study may be that the study population were composed of patients with kidney disease who had difficulty in controlling blood glucose and required a large amount of insulin.

Severe hypoglycemia has proven to be an independent risk factor for eGFR reduction and increased urinary albumin excretion, aggravating the occurrence and development of kidney disease. Currently, studies have shown that the incidence of hypoglycemic events in DKD patients during the use of intensive glycemic control measures is about 20%–40% [16–18]. In this study, a total of 52 (34.7%) T2DKD patients had experienced hypoglycemia. The analysis of the correlation between the occurrence of hypoglycemic events and the general situation of patients showed that the incidence of hypoglycemic events in T2DKD patients was mainly related to eGFR (*p*=0.003) and renal function grouping (*p*=0.007), indicating that patients with poorer kidney function were more prone to hypoglycemic events. The study also included the patients who were using combined oral medication and found that combined oral medication did not increase the risk of hypoglycemia.

We consider that these results of the study provide new evidence for the CSII treatment in the T2DKD population, helping to determine the appropriate insulin dosage and basal rate ratio, and further improve blood glucose control in T2DKD patients. The limitations of this study are mainly: Firstly, this is a retrospective study that only included T2DKD patients using CSII in this center, and the sample size is relatively small. Further prospective studies with larger sample sizes are needed to explore dosage setting strategies for CSII in the T2DKD population. Secondly, the study did not explore the long-term changes in insulin dosage with CSII use nor did it compare the insulin dosage of CSII with that of the MDI regimen. Further research is needed to support these findings.

## 5. Conclusions

In patients with T2DKD who received short-term CSII therapy, the insulin requirement declined with the decrease of eGFR, but the proportion of basic insulin did not change significantly. The dosage of insulin should be adjusted according to the level of eGFR in patients with T2DKD treated with CSII.

## Figures and Tables

**Table 1:** Clinical characteristics of patients with T2DKD among subgroups.

	Group G1 (*n* = 37)	Group G2 (*n* = 39)	Group G3 (*n* = 37)	Group G4–5 (*n* = 37)	*p* value
Gender (male/female)	24/13	22/17	22/15	23/14	0.878
History of hypertension	15 (40.5%)	25 (64.1%)	25 (67.6%)	33 (89.2%)	0.953
History of smoking	9 (24.3%)	11 (28.2%)	6 (16.2%)	7 (18.9%)	0.617
Family history of diabetes	16 (43.2%)	13 (33.3%)	15 (40.5%)	17 (45.9%)	0.727
Combination of OADs	24 (64.9%)	23 (59.0%)	10 (27.0%)^ab^	15 (40.5%)	0.004
Age (years)	54.65 ± 11.85	61.87 ± 10.91^a^	64.08 ± 7.62^a^	62.7 ± 9.55^a^	≤ 0.001
Duration of illness (months)	96 (42.132)	132 (72.180)	144 (84.240)^a^	180 (108.240)^a^	0.002
Waist circumference (cm)	89.5 ± 12.5	89.8 ± 12.5	85.5 ± 21.6	89.2 ± 9.6	0.865
HbA1c (%)	9.83 ± 2.42	9.65 ± 2.07	8.91 ± 1.89	8.38 ± 1.99^ab^	0.011
Fasting C-peptide (nmol/L)	0.44 ± 0.22	0.4 ± 0.25	0.43 ± 0.37	0.67 ± 0.53^bc^	0.010
UA (mmol/L)	386.64 ± 113.98	422.3 ± 121.1	448.05 ± 112.64	469.81 ± 77.89^a^	0.009
BMI (kg/m^2^)	24.93 ± 4.68	24.54 ± 3.36	24.89 ± 3.6	23.25 ± 3.47	0.196
Cr (μmol/L)	57.16 ± 15.57	83.49 ± 16.26^a^	135.97 ± 27.93^ab^	269.35 ± 109.53^abc^	≤ 0.001
eGFR (mL/min/1.73m^2^)	107.75 ± 13.1	78.15 ± 8.93^a^	44.25 ± 8.8^5abc^	21.21 ± 6.45^abc^	≤ 0.001
Uacr (mg/g)	409.57 ± 611.78	375.42 ± 715.23	1015.65 ± 1431.57	2950.97 ± 2493.87^abc^	≤ 0.001
LDL-c (mmol/L)	3.02 ± 1.48	3.22 ± 1.82	2.95 ± 1.26	3.03 ± 1.35	0.872
Duration of reaching blood glucose level (days)	5.46 ± 1.95	6.1 ± 1.73	5.76 ± 1.67	6.53 ± 1.99^a^	0.081

*Note:* HbA1c, glycated hemoglobin; Cr, serum creatinine.

Abbreviations: BMI, body mass index; eGFR, estimated glomerular filtration rate; LDL-C, low-density lipoprotein cholesterol; OADs, oral antidiabetic drugs; UA, uric acid; Uacr, urine albumin/creatinine ratio.

^a^The difference was statistically significant compared to the G1 group.

^b^The difference was statistically significant compared to the G2 group.

^c^The difference was statistically significant compared to the G3 group;

**Table 2:** Insulin dose characteristics in different subgroups of T2DKD.

Study groups	Number of cases	TDD		TDD kg^−1^		TBa kg^−1^		TBo kg^−1^		% TBa	
		At achievement	At the end of the study	At achievement	At the end of the study	At achievement	At the end of the study	At achievement	At the end of the study	At achievement	At the end of the study
Group G1	37	49.52 ± 13.74	47.87 ± 15.74	0.74 ± 0.20	0.73 ± 0.22	0.34 ± 0.12	0.32 ± 0.14	0.41 ± 0.11	0.40 ± 0.13	45.57 ± 8.33	44.03 ± 10.11
Group G2	39	43.88 ± 12.66	42.72 ± 12.29	0.67 ± 0.18	0.66 ± 0.18	0.30 ± 0.12	0.29 ± 0.12	0.37 ± 0.10	0.37 ± 0.10	45.91 ± 8.64	43.29 ± 12.14
Group G3	37	41.17 ± 15.22^a^	40.3 ± 15.91^a^	0.62 ± 0.21^a^	0.61 ± 0.22^a^	0.29 ± 0.10	0.27 ± 0.11	0.34 ± 0.15^a^	0.34 ± 0.15	47.40 ± 10.21	45.63 ± 10.13
Group G4-5	37	35.55 ± 18.26^ab^	36.2 ± 17.23^a^	0.58 ± 0.27^a^	0.59 ± 0.25^a^	0.26 ± 0.13^a^	0.26 ± 0.12^a^	0.33 ± 0.18^a^	0.33 ± 0.17^a^	45.22 ± 12.78	44.11 ± 11.92

*Note:* TDD, total daily insulin dose; TDD kg^−1^, total daily insulin dose per kilogram; TBa kg^−1^, total basal insulin dose per kilogram; TBo kg^−1^, total bolus insulin dose per kilogram; %TBa, the ratio of total basal insulin dose to total daily insulin dose.

^a^ *p* < 0.05 compared to group G1.

^b^ *p* < 0.05 compared to group G2.

**Table 3:** Insulin dose characteristics of Group A and Group B in patients with diabetic nephropathy.

		Group A (*n* = 74)	Group B (*n* = 76)	*p* value
TDD	At achievement	46.62 ± 13.41	38.36 ± 16.93	0.001
	At the end of the study	45.23 ± 14.22	38.25 ± 16.60	0.006

TDD kg^−1^	At achievement	0.71 ± 0.19	0.60 ± 0.24	0.003
	At the end of the study	0.69 ± 0.20	0.60 ± 0.23	0.013

TBa kg^−1^	At achievement	0.32 ± 0.12	0.27 ± 0.12	0.016
	At the end of the study	0.31 ± 0.13	0.26 ± 0.12	0.035

TBo kg^−1^	At achievement	0.39 ± 0.10	0.34 ± 0.16	0.017
	At the end of the study	0.38 ± 0.12	0.34 ± 0.16	0.047

% TBa	At achievement	44.69 ± 10.33	46.15 ± 11.17	0.330
	At the end of the study	44.55 ± 11.11	45.09 ± 11.30	0.583

*Note:* TDD, total daily insulin dose; TDD kg^−1^, total daily insulin dose per kilogram; TBa kg^−1^, total basal insulin dose per kilogram; TBo kg^−1^, total bolus insulin dose per kilogram; %TBa, the ratio of total basal insulin dose to total daily insulin dose.

**Table 4:** Comparison of insulin dose characteristics between the group of CSII alone and the group combined with OADs in patients.

		Group G1			Group G2			Group G3			Group G4		
		Treated with CSII alone (*n* = 13)	Treated with combination (*n* = 24)	*p* value	Treated with CSII alone (*n* = 16)	Treated with combination (*n* = 23)	*p* value	Treated with CSII alone (*n* = 27)	Treated with combination (*n* = 10)	*p* value	Treated with CSII alone (*n* = 22)	Treated with combination (*n* = 15)	*p* value
TDD	At achievement	47.87 ± 11.38	50.41 ± 15.01	0.441	47.14 ± 11.77	41.61 ± 13.01	0.712	37.10 ± 12.48	52.14J ± 17.12	0.241	35.14 ± 18.04	36.16 ± 19.19	0.692
	At the end of the study	47.25 ± 13.90	48.21 ± 16.93	0.225	46.15 ± 12.43	40.3 ± 11.87	0.592	36.08 ± 13.45	51.70 ± 17.10	0.322	36.23 ± 12.48	36.15 ± 12.88	0.602

TDD kg^−1^	At achievement	0.72 ± 0.16	0.76 ± 0.22	0.168	0.73 ± 0.21	0.63 ± 0.15	0.082	0.56 ± 0.16	0.80 ± 0.24	0.029	0.58 ± 0.29	0.58 ± 0.25	0.835
	At the end of the study	0.71 ± 0.19	0.74 ± 0.24	0.306	0.72 ± 0.22	0.61 ± 0.14	0.054	0.55 ± 0.17	0.79 ± 0.25	0.048	0.60 ± 0.26	0.58 ± 0.24	0.669

TBa kg^−1^	At achievement	0.30 ± 0.09	0.36 ± 0.13	0.488	0.33 ± 0.16	0.28 ± 0.08	0.009	0.27 ± 0.08	0.35 ± 0.13	0.056	0.23 ± 0.11	0.29 ± 0.14	0.325
	At the end of the study	0.29 ± 0.11	0.34 ± 0.15	0.363	0.33 ± 0.15	0.27 ± 0.09	0.032	0.25 ± 0.09	0.34 ± 0.14	0.060	0.24 ± 0.10	0.29 ± 0.15	0.187

TBo kg^−1^	At achievement	0.42 ± 0.09	0.40 ± 0.12	0.126	0.40 ± 0.09	0.35 ± 0.10	0.926	0.30 ± 0.10	0.44 ± 0.21	0.013	0.36 ± 0.21	0.29 ± 0.12	0.220
	At the end of the study	0.42 ± 0.11	0.40 ± 0.13	0.744	0.39 ± 0.11	0.35 ± 0.09	0.275	0.30 ± 0.10	0.45 ± 0.20	0.046	0.35 ± 0.21	0.30 ± 0.10	0.073

%TBa	At achievement	42.33 ± 8.07	46.45 ± 8.16	0.519	44.85 ± 15.34	44.35 ± 8.22	0.232	47.30 ± 9.28	46.41 ± 14.78	0.073	42.29 ± 14.83	49.10 ± 8.02	0.091
	At the end of the study	42.23 ± 11.90	45.43 ± 9.55	0.477	44.09 ± 15.11	43.01 ± 10.69	0.617	45.65 ± 9.03	44.34 ± 14.09	0.480	42.56 ± 13.13	47.64 ± 6.62	0.087

*Note:* TDD, total daily insulin dose; TDD kg^−1^, total daily insulin dose per kilogram; TBa kg^−1^, total basal insulin dose per kilogram; TBo kg^−1^, total bolus insulin dose per kilogram; %TBa, the ratio of total basal insulin dose to total daily insulin dose.

**Table 5:** Multiple linear regression of TDD kg^−1^ when achieving blood glucose target in T2DKD patients.

	Nonstandardized coefficient		Standard coefficient	*t*	Sig.	95.0% confidence interval of *B*	
	*B*	Standard error				Lower limit	Upper limit
Constant	0.343	0.075		4.576	≤ 0.001	0.195	0.492
HbA1c%	0.002	0.001	0.249	2.873	0.005	≤ 0.001	0.003
eGFR (mL/min/1.73 m^2^)	0.023	0.008	0.243	2.796	0.006	0.007	0.039

*Note:* Dependent variable, total insulin amount per kg weight when achieving the blood glucose target; TDD kg^−1^, total daily insulin dose per kilogram; HbA1c, %, glycated hemoglobin.

Abbreviation: eGFR, estimated glomerular filtration rate.

**Table 6:** Incidence of hypoglycemia events in T2DKD population with different renal function groups.

Indicator	Number of cases	Hypoglycemia	*p* value
*Renal function grouping*			*0.007*
Group G1	37	7 (19.0%)	
Group G2	39	10 (26.4%)	
Group G3	37	20 (54.1%)^a^	
Group G4-G5	37	15 (41.0%)	

Abbreviation: T2DKD, type 2 diabetic kidney disease.

## Data Availability

The data underlying this article will be shared on reasonable request to the corresponding authors.

## Supporting Information

Supporting Table 1 is a description of the four eGFR groups.
