# Supplementary material for: Insulin Requirement Profiles of Short-Term Continuous Subcutaneous Insulin Infusion Therapy in Patients With Type 2 Diabetic Nephropathy
Source: Int J Endocrinol. 2025 Mar 13;2025:8403917. doi: 10.1155/ije/8403917 (PMC11925604; doi:10.1155/ije/8403917)
Supplement: Supporting Information — Additional supporting information can be found online in the Supporting Information section. [file 8403917.f1.docx]

Supplementary Table 1 Grouping criteria

| Study group | eGFR(ml/min/1.73m^2^) | Number of cases |
| --- | --- | --- |
| Group G1 | ≥90 | 37 |
| Group G2 | ≥60 and ＜90 | 39 |
| Group G3 | ≥30 and ＜60 | 37 |
| Group G4-G5 | ＜30 | 37 |
